# Thoracic endovascular repair technique for the treatment of patent ductus arteriosus in an elderly patient: A case report

**DOI:** 10.1097/MD.0000000000013558

**Published:** 2018-12-10

**Authors:** Jung Hee Kim, Jong Hyun Baek

**Affiliations:** a Department of Thoracic and Cardiovascular Surgery, Daegu Veterans Medical Center; b Department of Thoracic and Cardiovascular surgery, College of Medicine, Yeungnam University, Daegu, Korea.

**Keywords:** elderly PDA, patent ductus arteriosus, PDA with calcification, thoracic endovascular aortic repair

## Abstract

**Rationale:**

Patent ductus arteriosus (PDA) ligation by open surgery is more difficult and dangerous in elderly patients than in infants. Nowadays, simple and safe interventional catheterization technology is used for the closure of uncomplicated isolated PDAs. Therefore, an alternative less invasive therapeutic option must be developed to treat complicated PDA.

**Patient concerns:**

A 53-year-old woman presented with gradually exacerbated dyspnea on effort and continuous cardiac murmurs.

**Diagnosis:**

Transthoracic echocardiography (TTE) and contrast-enhanced 3D computed tomography (CT) were performed and revealed a conically shaped large PDA with calcification.

**Interventions:**

We used a nontouch exclusion technique with thoracic endovascular repair (TEVAR) for the treatment of this rare complicated PDA. The patient had an adequate proximal landing zone, and a tapered stent graft (S&G, Bio 34–30 mm × 110 mm, Korea) was used.

**Outcomes:**

Aortography after stent graft implantation revealed complete occlusion of PDA and no endoleaks. The procedure was successful, and the patient showed no vascular or other complications during follow-up.

**Lessons:**

TEVAR is a less invasive solution for pathologies of the thoracic aorta, such as aortic dissection or aneurysm. TEVAR is an established, simple, and safe method for repairing the thoracic aorta and can be a new alternative to other transcatheter techniques for complicated PDAs in elderly patients.

## 1 Introduction

Currently, various newly developed, less invasive, and effective endovascular repair techniques are being commonly used and have an important role in the closure of patent ductus arteriosus (PDA).^[1–3]^ However, adult PDAs, which are characterized by abnormal shape, large size, or concomitant calcification or aneurysm, may not be suitable candidates for treatment using these technologies and can lead to critical complications. Considering the vulnerable tissue and abnormal morphology of adult PDAs, endovascular repair using a coil or an occluding device can result in complications such as device migration, rupture, and residual shunt.^[1,3]^ Thus, the nontouch exclusion technique, thoracic endovascular repair (TEVAR), should be considered for closing complicated adult PDAs to prevent severe complications.^[4]^ We present a case of complicated PDA in an elderly patient that was treated successfully with TEVAR.

## 2 Case report

A 53-year-old woman underwent consultation at a local clinic for arthralgia. Continuous cardiac murmurs were detected at the left upper sternal border region and dyspnea on effort that gradually exacerbated. She was referred to the cardiothoracic department at the university hospital in the same city. Her height and body weight were 153 cm and 67 kg, respectively. Chest radiography revealed increased pulmonary vascular shadow and cardiomegaly. Electrocardiography showed left ventricular hypertrophy. Transthoracic echocardiography (TTE) demonstrated left atrial (LA) and left ventricular (LV) dilatation. The LA volume was 85 mm, and the LV end diastolic dimension was 58 mm. Dilated ascending aorta with mild aortic regurgitation and mild mitral valve regurgitation were observed. In addition, TTE revealed the presence of a PDA with continuous flow observed from the descending aorta to the pulmonary artery (PA). The estimated pulmonary to systemic ratio (*Q*_p_/*Q*_s_) was 1.2.

**Figure 1:**
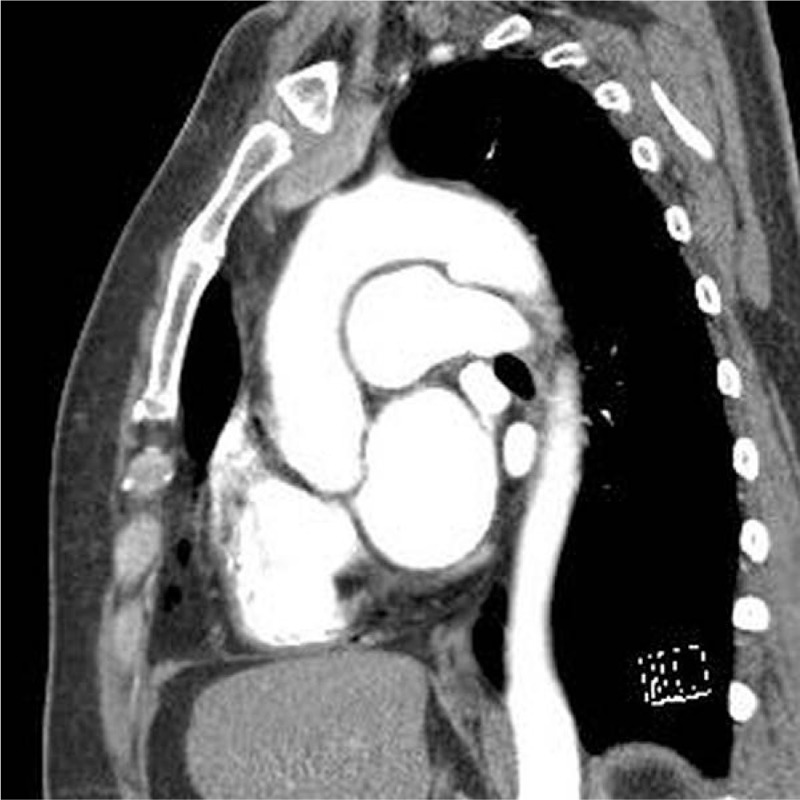
Preoperative three-dimensional computed tomography image of the aorta. Sagittal view showing communication between the aorta and pulmonary artery.

**Figure 2:**
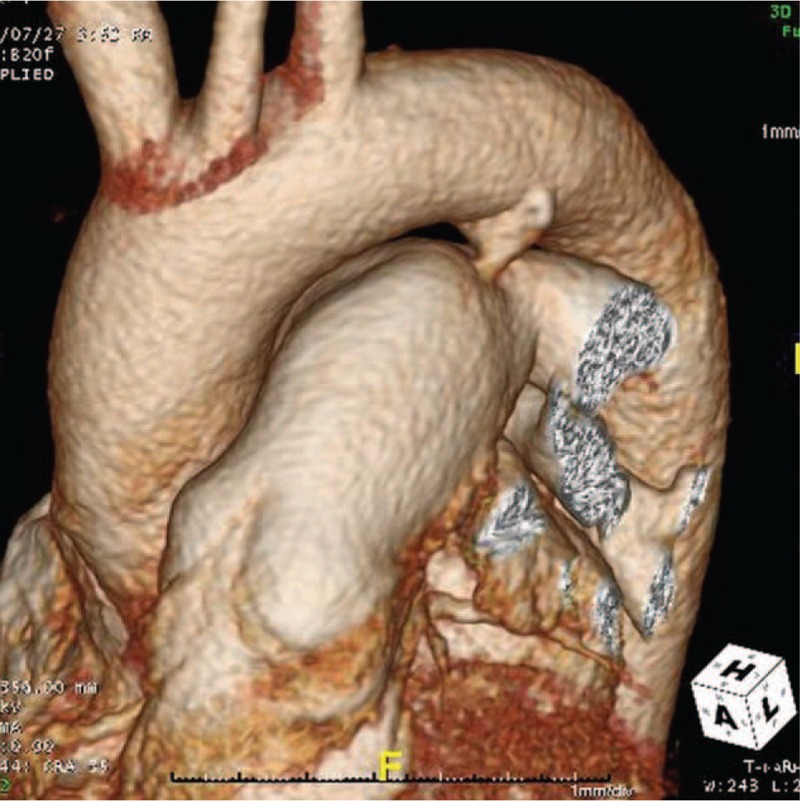
Preoperative three-dimensional computed tomography image of the aorta showing calcification on the aortic side.

Contrast-enhanced 3D computed tomography (CT) helped to determine the anatomical details around the PDA (Fig. 1). It revealed a conically shaped duct with calcification at the aortic end (Fig. 2). PDA with calcification at the aortic end was 14 mm in diameter, arising from the descending aorta, and was 30 mm distal to the left subclavian arterial (LSCA) orifice on the lesser curvature side.

PDA closure devices, such as the Amplatzer duct occluder (AGA Medical, Golden Valley, MN) or coil embolization, were deemed too risky for this patient. These percutaneous interventions have potential risks because friable tissue due to aneurysmal change, calcification, and the morphology of the PDA, may result in severe complications such as device migration, rupture, or residual shunt. Open surgery also carries a high risk because cardiopulmonary bypass (CPB) is required due to calcification. We decided to use a stent graft, which is usually used for treating thoracic aortic diseases.

TEVAR was performed in a catheterization laboratory under general anesthesia. Aortography before stent graft implantation demonstrated that most of the contrast agents ran into the PA through the PDA. A tapered stent graft (S&G, Bio 34–30 mm × 110 mm, Korea) was used. The patient had an adequate proximal landing zone, including the LSCA orifice. Half of the LSCA orifice was covered. A stent graft was successfully implanted. Aortography immediately after stent graft implantation revealed complete occlusion of the PDA. No endoleaks were observed. The procedure was completed in 75 minutes.

**Figure 3:**
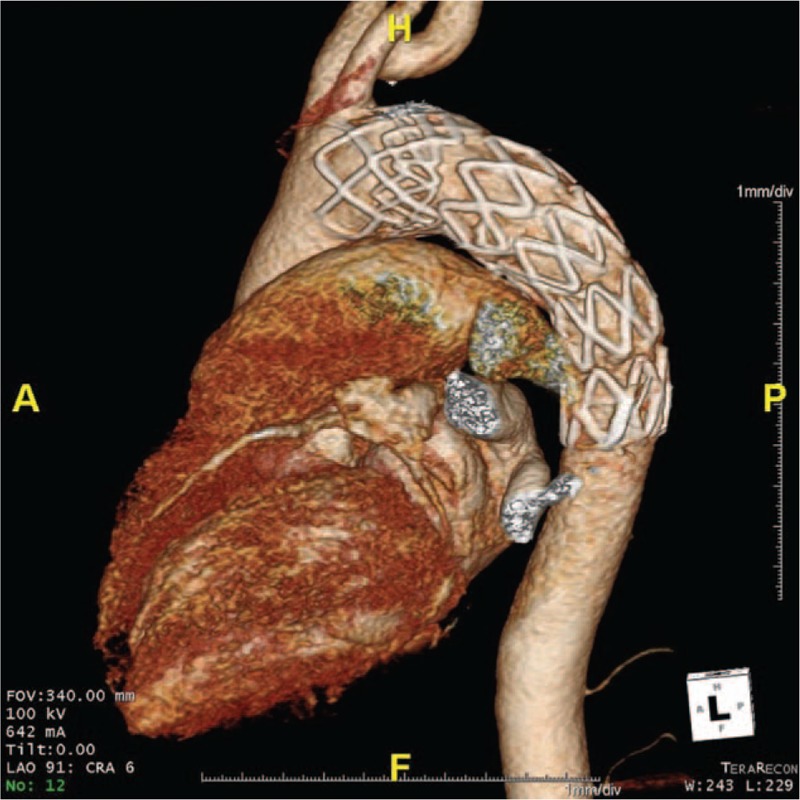
Postoperative three-dimensional computed tomography image of the aorta showing successful deployment of the stent.

The patient's postoperative course was uneventful and she was discharged on the 9th day after surgery. There were no complications, such as left arm claudication or vertebrobasilar insufficiency, either after the operation or during the follow-ups. CT was conducted at discharge (Fig. 3) and once a year after the procedure. Follow-up CTs have shown no other vascular complications such as endoleak or fistula, and the patient is doing well.

The Human Research Ethics Committee of Yeungnam University Hospital waived the requirement for approval as this is a single-case report. The patient provided written informed consent for publication of clinical details and images.

## 3 Discussion

The PDA in the present case was calcified, large in size, and had an abnormal shape, thus making treatment for PDA difficult and risky. Furthermore, the techniques already in use for repairing PDAs are not appropriate for this complicated case. Hence, another novel and secure method is necessary for this rare PDA, which we present in this study.

PDAs in the elderly are rare because most PDAs are detected and treated during childhood. If the duct remains in the elderly, the closure of PDA should be considered to prevent bacterial endarteritis, congestive heart failure, and pulmonary vascular disease.^[5]^

PDAs diagnosed during childhood can be treated by simple surgical ligation under thoracotomy or percutaneous catheterization, and treatments for childhood PDAs are relatively simpler and safer than those for PDAs in adults. PDA ligations or interventions in the elderly are more difficult and dangerous to perform than those in infants because of the characteristics of PDAs in adults. They are different from those in children owing to the calcification of the duct or around the aorta, aneurysms of PDAs, tissue friability, large size, and deformity.^[6]^

Treatments for PDA are surgical closure and percutaneous catheterization. The detailed options include endovascular occlusion with devices or coil embolization, and surgical correction with cardiopulmonary bypass or simple ligation. Less invasive and more effective percutaneous catheterization technologies are more commonly used for PDA closure, but surgical PDA closure in adults is still the treatment of choice for complicated PDAs unsuitable for endovascular intervention, which carries the risk of concomitant calcification, aneurysms, and cardiovascular or aortic diseases.^[7]^

Transcatheter procedures using a coil or an occluding device cannot replace surgery for closure of all complicated PDAs. The Amplatzer duct occluder has been employed for moderate-to-large ducts up to 12 mm in diameter,^[1]^ and coil embolization is usually performed for ducts smaller than 4 mm in diameter.^[2]^ Transcatheter intervention is less invasive than surgery, but the device or coil manipulation through the friable duct in the elderly has other types of risk. It may lead to severe complications. Calcification of the duct reduces the elasticity of the PDA and may lead to residual shunt or device migration after the procedure. In addition, the vulnerable duct tissue may lead to dissection or perforation.^[3]^

TEVAR is also a transcatheter method, as with coil embolization or device occlusion, but it is different due to its distinguishing features. For example, it does not require direct manipulation of the duct. This characteristic can prevent critical complications. Avoiding contact with the vulnerable tissue may reduce procedure-related complications, such as dissection or perforation, which are observed in other endovascular procedures. Another advantage is that duct exclusion by stent graft enables TEVAR to play an important role in inhibiting device migration or residual shunting for abnormally shaped or large size PDAs. These features give TEVAR a good opportunity to be a novel alternative to surgery or other percutaneous procedures, especially in case of abnormally-shaped or large, complicated PDAs with calcifications or aneurysms.^[4]^

Based on our experience, TEVAR is generally used in cases of complicated PDA in elderly patients under the following conditions: the diameter of the PDA is >12 mm; the PDA is abnormally shaped and is not cylindrical; presence of calcification or aneurysm around the PDA; and the presence of concomitant pathological conditions in the thoracic aorta. In the present case, we chose TEVAR as a new attempt for a complicated PDA, because the patient had a calcified, large, and deformed PDA. The patient expressed emphatic refusal to undergo surgery. The distance from the left subclavian artery to the duct was 30 mm. It was suitable to serve as a landing zone during TEVAR. Some studies reported the successful closure of calcified PDA using TEVAR with only a few complications in elderly patients.^[8,9]^

This established, simple, and safe approach can be a new alternative to other transcatheter techniques for complicated PDAs in elderly patients. TEVAR may be feasible for closing PDAs, particularly if other devices are not suitable. In addition, PDA closure using stent grafts is an effective measure for thoracic aortic diseases, such as aneurysm and dissection, and is one of the approved applications for TEVAR.^[10]^

In conclusion, as observed in this case, the nontouch exclusion technique, TEVAR, when smartly used, can have various applications and can be an additional solution for complicated PDAs in adults. Surgery involving this technique should be performed with careful follow-up monitoring considering a large enough landing zone, confirmation of cerebral blood flow, and postoperative endoleakage.

## Author contributions

**Conceptualization:** Jung Hee Kim, Jong Hyun Baek.

**Funding acquisition:** Jong Hyun Baek.

**Supervision:** Jong Hyun Baek.

**Writing – original draft:** Jung Hee Kim.

**Writing – review & editing:** Jung Hee Kim.
